# Microglial Dynamics During Human Brain Development

**DOI:** 10.3389/fimmu.2018.01014

**Published:** 2018-05-24

**Authors:** David A. Menassa, Diego Gomez-Nicola

**Affiliations:** Biological Sciences, Faculty of Natural and Environmental Sciences, Southampton General Hospital, University of Southampton, Southampton, United Kingdom

**Keywords:** microglia, brain development, glial cells, proliferation, human brain, neurodevelopment

## Abstract

Microglial cells are thought to colonize the human cerebrum between the 4th and 24th gestational weeks. Rodent studies have demonstrated that these cells originate from yolk sac progenitors though it is not clear whether this directly pertains to human development. Our understanding of microglial cell dynamics in the developing human brain comes mostly from postmortem studies demonstrating that the beginning of microglial colonization precedes the appearance of the vasculature, the blood–brain barrier, astrogliogenesis, oligodendrogenesis, neurogenesis, migration, and myelination of the various brain areas. Furthermore, migrating microglial populations cluster by morphology and express differential markers within the developing brain and according to developmental age. With the advent of novel technologies such as RNA-sequencing in fresh human tissue, we are beginning to identify the molecular features of the adult microglial signature. However, this is may not extend to the much more dynamic and rapidly changing antenatal microglial population and this is further complicated by the scarcity of tissue resources. In this brief review, we first describe the various historic schools of thought that had debated the origin of microglial cells while examining the evidence supporting the various theories. We then proceed to examine the evidence we have accumulated on microglial dynamics in the developing human brain, present evidence from rodent studies on the functional role of microglia during development and finally identify limitations for the used approaches in human studies and highlight under investigated questions.

## Introduction

Microglia are resident macrophages of the mammalian central nervous system (CNS) with a plethora of functions including innate immunity and neuroprotection (1, 2), synaptic pruning (3), and the phagocytosis of cellular debris (4). Microglia also have a dark side, suggested to be indirectly involved in pathologies such as schizophrenia (5) and more directly in Alzheimer’s disease (6). They play a central role in driving neuroinflammation and also coordinating this response with the systemic compartment and with other glial cells including astrocytes (7). A careful characterization of human microglial morphology, transcriptome, regional heterogeneity, and turnover in the adult human brain is emerging greatly due to the increased availability of appropriately preserved postmortem tissue. In the adult human cortex, for example, microglial cells acquire a ramified morphology with processes surveying the neighboring environment and an amoeboid morphology with retracted processes when they are phagocytosing the debris of dying neurons or synapses or reacting to a foreign body (8). Adult human microglia are enriched for genes associated with the inflammasome, cell-adhesion, and immune modulation among others and these characteristic profiles have been helpful in defining the differences and similarities with the better characterized model of murine microglia (9, 10). Adult human microglia also demonstrate regional heterogeneity with lower densities in the gray matter compared with the white matter and substantially lower densities in the cerebellar cortex, for example, compared to regions with higher densities such as the substantia nigra (11, 12). Adult human microglia renew slowly at a median rate of 28% per year and at any given moment, only 2% of microglia are thought to be proliferating (13, 14). It is suggested that the spatiotemporal coupling between proliferation and apoptosis maintains microglial density throughout life (14). In sum, though our understanding of the human adult microglial population has progressed tremendously, we know very little about these cells during human brain development. In this short review, we first present a brief history of the discovery of microglia and then examine the various schools of thought that had debated the origins of these cells. We then proceed to present an overview of what is known about microglia during human brain development (see Table 1 for a summary of the main studies on microglia in developing human tissue) and examine how rodent studies have helped elucidate some of the key functions of microglia during brain development. We conclude by discussing the challenges associated with the utilized approaches in human studies.

**Table 1:** A summary of main microglial topography studies in embryonic and fetal human tissue.

Reference	Year	Age (weeks)	Sample size	Anatomical areas	Markers used	Findings and conclusions
Rydberg (24)	1932	38.0	2	Cerebrum, cerebellum, spinal cord	Silver carbonate	First study on microglial cytogenesis in human fetuses and newborns. Proponent of the neuroectodermal theory, this study suggests that any glial cell could originate from amoeboid glia located along the ependymal matrix
Von Santha (23)	1932	24.0 and 29.0	2	Basal ganglia, telencephalon, white matter tracts, subcortex, medulla	Silver carbonate	Microglia are ramified in the basal ganglia, the telencephalon, and the medulla. Plump branched cells are observed in the subcortex and prospective white matter tracts. No globose forms are reported
Juba (25)	1933	10.0–34.0	7	Cerebrum, cerebellum	Silver carbonate	Microglia are amoeboid early in development and ramify when they become differentiated. Microglia enter the developing brain from mesodermal elements closely associated with the vasculature. Microglia are genetically related to monocytes
Kershman (76)	1939	8.0–29.0	22	Cerebrum, cerebellum, spinal cord	Silver carbonate, H&E	The youngest form of microglia is amoeboid first before they ramify into a mature form. Amoeboid cells enter into the brain from specific locations known as fountains located around the choroid plexus, the meningeal compartment, and blood vessels. First evidence of microglia is in the rhombencephalon at 8.0 weeks. Once in the developing brain, amoeboid microglia acquire pseudopods as they penetrate deep into the brain matter and eventually acquire a final stable ramified phenotype
Choi (62)	1981	6.0–22.0	53	Cerebrum, cerebellum, spinal cord	H&E; Toluidine Blue	6.0–12.0 weeks: hematogenous cells are found in the cerebellum, spinal cord, and cerebrum. These become rarer as the fetus gets older. The neuropils of embryos/fetuses contain macrophage-like cells inside and outside of blood vessels. Subpial, perivascular, and perineuronal regions of neural parenchyma contain small cells with fusiform nuclei, some with elongated nuclei and some with rod-shaped/oblong nuclei
Fujimoto et al. (66)	1989	7.5–26.0	7	Cerebrum	NDP-ase	Vascularization in the cerebral cortex is apparent by 9.0 weeks, vessels invade the pallium. Amoeboid NDP-ase cells appear around the vessels. The MZ has poorly ramified microglia. By 11.0 weeks, amoeboid cells present in the matrix and subcortical layers. Highly ramified NDP-ase cells are seen in layer I, the subcortex, and cortical mantle between 22.0 and 26.0 weeks
Hutchins et al. (77)	1990	13.0–24.0	22	Frontal cerebrum	RCA-1; EBM-11	Densest RCA-1 and EBM-11 cells are present in the germinal matrix at 13.0 weeks. Microglial morphology is variable between the gray and white matter between 13.0 and 18.0 weeks (amoeboid versus intermediate) and less variable and ramified across the mantle and white matter between 19.0 and 24.0 weeks
Esiri et al. (86)	1991	18.0-term	15	Telencephalon	EBM-11; HLA-DR	Amoeboid cells are present in the germinal matrix at high densities throughout this temporal window. Ramified microglia in gray and white matter are only seen from 35.0 weeks. Very little reactivity to HLA-DR is detected throughout
Gould and Howard (80)	1991	13.0–27.0	19	Cerebral white matter	A1ACT; MAC387	Macrophage clusters positive for A1ACT accumulate in prospective white matter hotspots including the periventricular areas, corpus callosum, the junction between the external and internal capsule, the optic tract, and the thalamus. Increased densities followed a caudo-rostral pattern with more rostral areas showing microglial fountains after 22.0 weeks
Hutchins et al. (90)	1992	18.0–24.0	6	Cervical spinal cord	RCA-1; EBM-11	RCA-1/CD68-positive amoeboid microglia are seen in the white matter and only RCA-1 but CD68 negative ramified microglia are seen in the gray matter
Wierzba-Bobrowicz et al. (43)	1995	7.0-term	47	Mesencephalon	RCA-1; Anti-Ferritin	RCA-1/Ferritin-positive amoeboid cells are observed in the mesencephalon between 16.0 and 40.0 weeks
Rezaie et al. (82)	1997	16.0–22.0	7	Frontal telencephalon	CD11b; CD68; CD64; CD45; RCA-1; BSB-4	Fetal RCA1 microglia co-occur with highly vascularized ICAM-1 positive vessels within the developing brain
Andjelkovic et al. (63)	1998	4.5–13.5	14	Telencephalon, diencephalon, rhombencephalon	RCA-1; Tomato Lectin; CD68	At 5.5 weeks, CD68 and lectin positive cells are observed in the cerebellum and medulla. Between 6.0 and 7.5 weeks, first blood vessels in telencephalic wall and CD68/lectin cells increase in density in line with vascularization. Vascularization by CD34 tagging is prominent first caudally (cerebellum and diencephalon), then rostrally eventually. At 7.5 weeks, ramified microglia are detected in the cerebellum only among amoeboid cells. Between 8.0 and 9.0 weeks, clusters of microglia are observed in diencephalon. Ramified microglia are also observed in the MZ and IZ and none in CP. By 11.0–13.0 weeks, ramified microglia are detected in all layers of the cortex. Two classes of cells are identified here lectin positive and CD68/lectin positive
Wierzba-Bobrowicz et al. (12)	1998	8.0–22.0	72	Mesencephalon, cerebellum, frontal lobe	RCA-1; Anti-Ferritin; HAM56	Amoeboid microglia are increased in all structures as gestation progresses. White matter ramified microglia are highest in density by 22 weeks in the mesencephalon and the lowest in the white matter of the cerebellum
Rezaie et al. (31)	1999	7.0–15.0	9	Spinal cord	RCA-1; CD68; HAM56; CD11b; CD45; CD64	Microglia present are present in the spinal cord from 9.0 weeks and enter from the meninges/connective tissue unlike the cerebrum where they migrate out from the germinal matrix toward the cortical surface
Rezaie et al. (75)	2005	12.0–24.0	45	Telencephalon	RCA-1; CD68; MHC-II	12.0–14.0 weeks of gestation, 2 populations are detected: CD68+/RCA-1+/MHC-II− amoeboid cells aligned within the subplate and RCA-1+/CD68−/MHC-II- cells in the MZ and lower CP that progressively ramify within the subplate. Microglia are absent from the germinal layers and IZ. From 14.0 to 15.0 weeks, MHC-II+/CD68+ cells in the germinal layers and corpus callosum appear and further populate the lower telencephalon from 18.0 to 24.0 weeks
Billiards et al. (84)	2006	22.0–37.0	9	Cerebral white matter	Tomato lectin; CD68; MHC-II	Ramified microglia are only detected in the prospective white matter at 22.0 weeks then intermediate or amoeboid forms are seen between 25.0 and 37.0 weeks in similar areas
Monier et al. (60)	2006	5.0–24.0	31	Diencephalon, telencephalon	IBA-1; CD68; CD45; MHC-II; LN3; HLA-DR	By 5.5 weeks, accumulation of IBA-1 microglia near the meninges and choroid plexus before blood vessel formation in the brain parenchyma. Early entry along the di-telencephalic fissure of amoeboid cells from within the choroid plexus and meninges is reported. These are fetal macrophages reported by Choi and Andjelkovic et al. (1998). IBA-1/CD34 associated cells are observed at 10.0 weeks in the white matter
Monier et al. (61)	2007	4.5–24.0	33	Telencephalon	IBA-1; CD68; CD45; MHC-II; LN3; HLA-DR	Microglia express IBA-1 at 4.5 weeks. Amoeboid cells are present in the lumen and leptomeninges then. Entry into the brain is through the ventricles, whereby microglia assume eventually tangential and radial migration routes. Other route is from the pia to the MZ. Microglia are ramified by 22.0 weeks in the cortical mantle, amoeboid in the IZ, and grouped in clusters between 22.0 and 23.5 weeks
Verney et al. (73)	2010	20.0–32.0	4	Telencephalon, diencephalon	IBA-1	Accumulation of IBA-1 microglia in clusters is seen in the white matter (centrum semiovale)
Cho et al. (89)	2013	15.0–25.0	9	Brainstem, olive, gray matter, white matter, hippocampus	H&E; HLA-DR2; CD68	CD68 positive cells are evident in the floor of the fourth ventricle, the pons and olive at 15.0–16.0 weeks, accumulating in and around the hippocampus at 22.0–25.0 weeks. At both stages, the accumulation of these cells was evident in the optic tract and the anterior limb of the internal capsule. GAP-43 developing axons are not associated with CD68 cells
Mildner et al. (88)	2016	11.0–35.0	4	Cerebellum, white matter, cortex	P2Y12; CD68; IBA-1; HLA-DR	Clusters of microglia are detected between the lateral ganglionic eminence and the caudate nucleus, as well as in nerve fibers spanning the caudoputamen. Two smaller clusters of P2Y12 microglia are also visible in the thalamic region and the optic tract. All P2Y12 microglia express IBA-1. Non-clustered microglia have moderate CD68 immunoreactivity but strong P2Y12 reactivity. P2Y12 amoeboid myeloid cells are seen in the choroid plexus in an 11.0-week fetus. Choroid plexus cells are positive for IBA-1, but show very low immunoreactivity against P2Y12

*CP, cortical plate; IZ, intermediate zone; H&E, hematoxylin and eosin; MZ, marginal zone*.

## On the Discovery of Microglia

In 1899, Franz Nissl, a German neuropathologist, reported on the presence of rod-shaped cells in human brain cases of syphilitic paralysis, a severe neuropsychiatric condition characterized by eventual dramatic cerebral atrophy (15). This was perhaps one of the earliest reported descriptions of rod-shaped/bipolar microglia, thought to correspond to an intermediate phenotype of cells. Decades later, Kreutzberg reported on the response of microglial cells to injury in a rodent model of facial nerve axotomy (16). Unlike Nissl’s more general observation, Kreutzberg was specifically investigating how the microglial population reacted to injury and demonstrated a marked increase in proliferation in rodents. Mononuclear phagocytes of the CNS such as microglia and macrophages were known as the mesoglia, being of mesodermal origin, and this classification distinguished them from astrocytes and oligodendrocytes that are of neuroectodermal origin (17). In 1913, Cajal optimized Camillo Golgi’s black reaction method by using the gold chloride sublimate to enhance the visualization of non-neuronal elements of the CNS that the first morphological description of the microglial cell body emerged (18). Cajal identified cell bodies without processes differentiating them from neurons and astrocytes. Due to limitations in his method, he could not observe the ramifications emanating from microglial cells. Subsequently, Pio Del Rio Hortega optimized Cajal’s histochemical method and used silver carbonate to selectively label and visualize the cell body and the ramified morphology of these cells (19). He also incepted the term “microglia” in 1919 and provided a thorough morphological and functional characterization of these cells from development to injury, providing seminal findings that settled long lasting disputes over the developmental lineage of microglia (20, 21) [for translations of Hortega’s work, see Ref. (22)]. In 1932, the first studies on microglia in human developmental tissue emerged (23–25). After Hortega and these initial studies, the investigation of microglial cells plunged into a long hiatus and received little attention from the neuroscience community, to be re-visited from the mid 1980s largely due to the technical advances of immunohistochemistry, with studies on the origin and identity of brain macrophages by Perry et al. (26) or Hickey and Kimura (27). Since then, the study of microglia has developed quickly and nowadays is one of the most active fields of neuroscience.

## The Origins of Microglia

**Figure 1:**
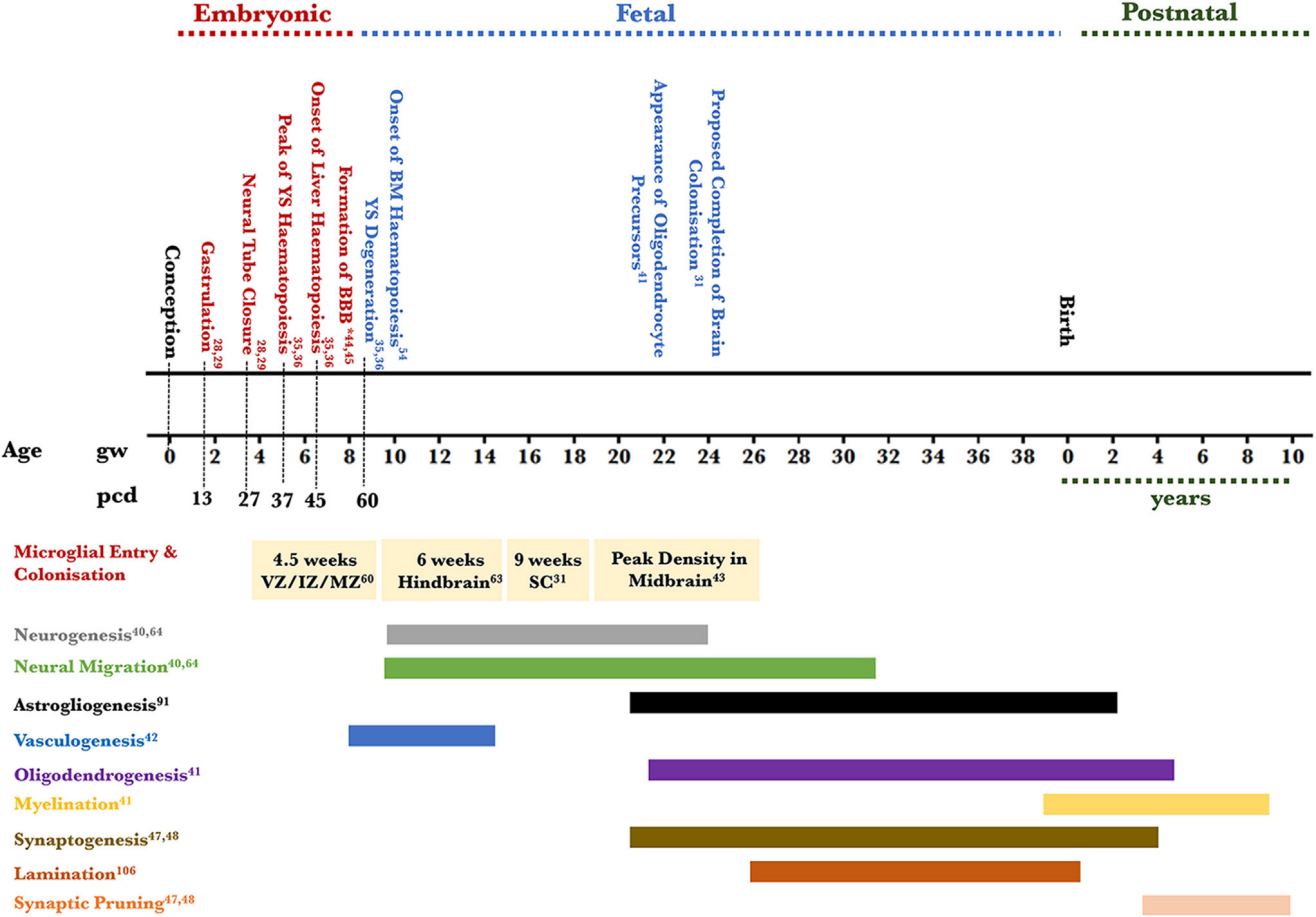
Timeline of human microglial development. This figure places microglial entry into developing brain areas in the context of the approximate co-occurrence of cortical developmental events. Microglia are detected in the amoeboid state in the telencephalic ventricular, intermediate, and MZs and not the cortical plate at 4.5 weeks of gestation; microglia are detected in the ramified state in the hindbrain by 6 weeks; amoeboid microglia are detected in the SC by 9 weeks of gestation; the density of amoeboid microglia is highest in the mesencephalon (midbrain) by 22 weeks of gestation. The relevant references to each process are included (28, 29, 31, 35, 36, 40–45, 47, 48, 54, 60, 63, 64, 70, 91, 106). *Two barriers are included here: the BBB proper at the level of the endothelium of the cerebral blood vessels and the blood–CSF barrier at the level of the choroid plexus epithelial cells. The CSF–brain barrier is detected as early as 16 days through the appearance of strap junctions and is not shown on the timeline. Abbreviations: BBB, blood–brain barrier; BM, bone marrow; CSF, cerebrospinal fluid; IZ, intermediate zone; MZ, marginal zone; PCD, post conceptional days; GW, gestational week; SC, spinal cord; YS, yolk sac.

A critical phase in human embryonic development is gastrulation whereby the blastula becomes organized into a trilaminar structure that will yield out all the organs. The endoderm, forms the gut lining; the mesoderm generates the circulatory, cardiovascular and lymphatic systems; and the ectoderm generates both skin and the neuroectoderm, from which the nervous system develops (28, 29). Microglial origins have been a subject of debate and two dominant theories argued whether these cells originated from precursors in the neuroectoderm or the mesoderm. The neuroectodermal theory argues that microglia originate in the neurepithelium from pluripotent glioblasts and are, therefore, akin to astrocytes and oligodendrocytes in lineage (17, 30, 31). *In vitro* evidence of rodent neuroepithelial or astroglial cultures generating microglia supported this theory (32). Additionally, when bacteriophage lambda transgenic mice bone marrows (BMs) were transplanted into recipient murine neonates and adults, *in situ* hybridization and immunohistochemistry showed that the donors’ BM-derived macrophages did not contribute to the adult microglial population arguing that microglia could be neuroectodermal in origin (33). The second theory argues that microglia originate in the yolk sac (YS) from hematopoietic stem cell progenitors akin to primitive macrophages and migrate to the brain rudiment as early as the fourth week of gestation in humans, the closure of the caudal neuropore occurring by 27 days post-conception [(17, 20), see also Figure 1]. Briefly, between the 15th and the 40th gestational days, the YS is the main hematopoietic organ and localized thickenings showing prospective blood islands are visible in the mesoderm by the 16th day and the peak of hematopoiesis is thought to be the middle of the fifth week of gestation (34–36). Rare histological reports demonstrate the occasional presence of macrophages prior to the onset of hematopoiesis in the yolk-sac by day 13, notably in the trophoblastic mesenchyme (37). By the sixth week of gestation, hepatic colonization by hematopoietic stem cells initiates subsequent hematopoiesis (38). Bone-marrow development does not occur until the 10th week of gestation, and it is well-established that it is not a hematopoietic organ until much later in term, and by the 50th post-conceptional day, YS hematopoiesis will have substantially decreased (34, 38, 39).

Evidence for the mesodermal theory comes from early studies showing the expression of macrophage markers in microglial cells such as FcγRI and CD45 suggesting an extra-cerebral origin and a shared ontology with macrophages within the mononuclear phagocytic system (49). In contrast, rodent studies have provided compelling evidence on the ontology of microglial cells. In mice, RUNX1^+^ (Runt-related transcription factor 1 is used as a marker for precursor myeloid cells in the extra-embryonic YS) progenitors appear prior to embryonic day 8 in the YS and generate the entire microglial adult population independently of any bone-marrow derived contribution (50). These precursors are thought to be c-KIT^+^ stem cells that eventually develop into mature CD45^−^/c-KIT^−^/CX3CR1^+^ cells in a PU-1, IRF-8, and colony stimulating factor 1 (CSF-1R)-dependent manner before they acquire their identity as microglial cells upon invading the brain rudiment (50–52). In the human YS and mesenchyme sampled between 4 and 6 gestational weeks (gw), two populations of cells with different phenotypes have been distinguished: the majority carried both macrophage-associated (RFD7^+^) and monocyte-associated markers (UCHM1^+^) with no detectable HLA-DR antigen, and the minority expressed class II (HLA-DR and -DP) and no RFD markers, alluding to the possibility of distinct signatures in these populations prior to BM development (53). Overall, it is highly likely that human microglia derive from primitive YS macrophages similarly to rodents. This is an early process that precedes hepatic and BM hematopoiesis (54). Subsequently, mononuclear phagocytes issued from YS hematopoiesis colonize the various organs including the human brain, akin the model proposed for mouse microglial development (55).

## Microglial Population of the Developing Brain

The human developing forebrain, midbrain, and hindbrain become identifiable following the closure of the neuropore by 27 post-conceptional days (56–59) (see Figure 1). The first wave of entry of microglia into the developing brain predates the peak of neurogenesis and neuronal migration in the telencephalon (40, 60–66). Neural progenitors in the various regional germinal neuroepithelia along the ventricular zone (VZ) proliferate between 8 and 28 gw and migrate toward the cortical plate between 9 and 38 gw (40, 65). The pattern of migration predicts the identity of the neuron in the developing cortex. Generally, excitatory projection neurons originating in the VZ/subventricular zone (SVZ) migrate radially to the cortical plate (64, 67) while inhibitory GABA-ergic neurons originating in the VZ/SVZ and the ganglionic eminence migrate tangentially to the cortical plate before undergoing radial migration in the intermediate zone (IZ) though this does not appear to be universal as a subclass of interneurons migrates radially (68–71). Subsequent to assuming their cortical positions and acquiring their identity, synaptogenesis (26 gw onward) ensues to establish connectivity across the developing brain and this process carries on beyond birth (65, 72). Some, thought to be mesoderm-derived cells, make their route to the developing brain very early and three microglial entry routes have been described: the leptomeningeal route, the ventricular lumen route and the choroid plexus route. In the telencephalic wall, amoeboid microglial cells are detected in the ventricular lumen and VZ as early as gw 4.5. Concomitantly, amoeboid microglia also accumulate in the superficial marginal zone (MZ) presumed to enter from the pial surface and by 5.5 gw some amoeboid cells are also presumed to enter from the choroid plexus into the parenchyma of the thalamic eminence. These cells are positive for ionized-calcium binding adaptor protein 1 (IBA-1) and are thought to be undifferentiated immature microglial cells (31, 60–63, 73). However, whether their transcriptional signature is already defined at that stage remains unresolved as some could belong to the macrophage lineage as there is some positivity for CD68, HLA-DR, and CD45. The density of microglial cells at the areas of entry during development does not correlate with their preponderance in areas of the adult brain. Therefore, it can be assumed that the cells would be migrating and proliferating or vice-versa (26). By the eighth gw and as the cortical plate thickens, intermediate rod-shaped microglial cells appear in the MZ and their density increases in the VZ, SVZ, IZ, and the subplate (74–77). Cells particularly in the IZ are suggested to be proliferating between 8 and 12 gw. By the 10th gw, and as fetal blood vessels appear in the telencephalic wall, some amoeboid microglia seem closely associated with the endothelium. Between the 9th and until the end of the 13th gw, proliferating amoeboid microglia aligned along the subplate-cortical plate boundary assume this location and do not enter the cortical plate well beyond its resolve by the third trimester (75, 78). These cells are suggested to support thalamocortical synaptic development (79). Between the 14th and 17th gw, already proliferated microglial cells cluster near or within the white matter at the junction between the thalamus and the internal capsule, the optic tract, and the junction between the internal capsule and the cerebral peduncle and later clusters are observed in the corpus callosum and the periventricular hypothalamus (60, 75, 80). By the 18th gw, intermediate microglia are suggested to migrate radially within the cortical plate to eventually develop into ramified gray matter microglia. Tangential migration occurs within the IZ yielding white matter microglia though interestingly, studies on quail retina suggest that initially, microglial cells migrate tangentially along the various brain layers then assume a radial migration pattern to reach the deep brain parenchyma (81). Microglial cells with various morphologies (intermediate or ramified) populate the cortical mantle and are distributed differentially across the various layers (60, 82, 83). Finally, between the 19th and 30th gw, tangentially oriented microglial packed clusters with an intermediate rod-shaped morphology are reported in white matter tracts such as the corpus callosum and the external capsule. Their density decreases after the 35th gw (73). It is interesting to note that the onset of myelination coincides with the temporal window during which these cells increase in size and proliferate. In humans, the appearance of oligodendrocyte precursors dates to the 22nd gw with myelination commencing when these cells differentiate and become functional from the 28th gw (31, 41). It is, therefore, tempting to speculate that increased microglial presence during this time could be phagocytic during the onset of myelination particularly as some neurons will have migrated into the white matter and would, therefore, need to be cleared. The evidence for this comes from the activated shape of these microglia in these regions within 22–30 gw (84). Furthermore, various authors have also alluded to this temporal window as being when white matter is most susceptible to injury (60, 73, 85). Therefore, increased microglial presence could be seen as protective during this phase. Evidence for this hypothesis comes from pre-term fetal tissue assessed for white matter injury (periventricular leukomalacia) where abnormally high densities of activated microglia are seen in the same regions as the aforementioned clusters (73). By the end of gestation, ramified microglia can be seen across the cortical layers and the white matter shows a higher density of cells compared to the gray matter (86).

Very few studies have focused on characterizing microglial populations in the developing midbrain, cerebellum, and spinal cord (SC). In the cerebellum, its molecular and granular layers are apparent by the 12th gw (87). Ramified microglial cells have been detected as early as 7.5 gw (63) and as the telencephalic wall thickens, very few amoeboid microglial cells remain in the cerebellum (88). It has been suggested that the caudorostral appearance of the vasculature (cerebellum to telencephalon) coincides with increases in microglial densities (12, 42, 63). Much less is known about the mesencephalon (future midbrain) though a couple of studies have highlighted that microglial ramified densities reach a record high during development in this area in comparison with other areas (12, 43, 89). Finally, in the SC, microglia are suggested to colonize the gray and white matter by the ninth gw (31). Their pattern of migration into the cord is thought to follow an outside-in pattern (from the meninges into the gray/white matters) (31). Toward 18–24 gw, SC gray matter microglia are ramified while white matter microglia are amoeboid (90).

Overall, microglial cells enter the developing brain very early preceding neurogenesis, neuronal migration, and gliogenesis in the regional neuroepithelia (40, 91). As development is a highly dynamic and metabolically demanding period, it is likely that apoptosis is coupled with proliferation as has been documented in the cortical subplate for example at the end of the first trimester (92). It is also interesting to consider the human blood–brain barrier (BBB) across cerebral endothelial cells and the CSF brain barrier across the neuroependyma, which shows mature tight junctions as early as the seventh gw as demonstrated by freeze-fracture studies on human embryos of that age (44, 45, 93, 94). Additionally, Gröntoft (and later confirmed by experiments on rabbit fetuses) clearly demonstrated that trypan blue in a 9 gw fetus did not reach the brain if injected in less than 10 min from fetal placental separation (cesarean section delivery) indicating an intact BBB (95). Thus, this may suggest that microglial populations could be expanding as a population from residing cells beyond the closure of the BBB. Furthermore, knowing that some areas of the brain develop much later than others (the cerebellar primary fissure is only visible from the 12th gw, for example) and after BBB closure has been completed, investigating microglial dynamics in this area may shed light on why the adult human cerebellum may contain much less microglial cells in comparison with gray and white matter regions.

## Functional Roles of Microglia During Brain Development

The early colonization of the developing brain by microglia antecedes neurogenesis, neuronal migration, and many other cellular processes (see Figure 1) suggesting that these cells are very likely to play pivotal roles in mediating the normal occurrence of some of these events. Multiple lines of functional evidence, predominantly from rodent studies, support this. First, maternal administration of liposomal clodronate to deplete fetal microglia or a maternal immune challenge to activate microglia affect the size of the neuronal precursor pool in the SVZ during the late stages of cortical neurogenesis (96). This has been demonstrated in the rat fetus and highlights the importance of the phagocytic role of microglia in restricting the number of neuronal precursors during development. Furthermore, microglia are thought to promote the apoptosis of Purkinje cells (through superoxide anion signaling during late cerebellar development) in the mouse (97), granular cells in the rat hippocampus (98), and ganglionic cells in the avian retina (81). Second, the transient blockade of CSF-1R at the onset of yolk-sac microglial progenitor production (E6.5) in the mouse embryo alters the normal positioning of a subclass of cortical interneurons (Lhx6) and causes aberrant dopaminergic axonal outgrowth (99). This supports the role of microglial cells in the migration of some cortical interneurons to their correct laminar position and provides novel evidence of the role of these cells in axonal outgrowth. Third, the embryonic absence of microglia in PU.1 knock-out mice is associated with a decreased rate of cortical astrocyte differentiation, suggesting an association between these two cell types (46). In fact, a close association between GFAP-vimentin positive cells and microglia has been already documented in the ninth gw human SC though the functional implication of this has not been elucidated (31). Fourth, challenging the immune system of pregnant dams (by lipopolysaccharide injection at E15.5) or mutating part of the microglial TREM2 signaling pathway results in defasciculation of the corpus callosum in mouse embryos (100). Furthermore, microglia are associated during development with axonal tracts, and this has been repeatedly shown in gestational human tissue (31, 60, 73). Hence, these findings suggest a role of microglia in axonal pruning and the support of myelination. Finally, fractalkine receptor (CX3CR1) knock-out mice have delayed synaptic pruning demonstrated by a transient excess of dendritic spines concomitant with decreased microglial numbers during development. Functionally, these mice had also reduced synaptic efficiencies in hippocampal long-term potentiation (101). Thus, microglial cells have also a pivotal role during development in synaptic refinement.

Altogether, microglial cells participate actively in various processes during brain development. Functional human studies are very difficult to conduct. However, and as previously discussed, histopathological studies on human embryonic and fetal tissues concur about the distribution of microglial cells in association with some key developmental processes including neuronal migration, white matter tract formation, vasculogenesis, as well as astrocyte formation and synaptic pruning.

## Limitations of Existing Methods and Future Prospects

Postmortem studies on human tissue are invaluable for investigating the antigenic features and morphology of microglial cells across various developmental ages. The identified studies in this review give us a qualitative insight into microglial dynamics during development. However, there are multiple caveats. First, though the total number of cases investigated in each study varied between *n* = 4 and *n* = 47 cases, it is extremely difficult to interpret the results when a single case is used to generalize over one gestational age. This is mainly because tissue is scarce. Additionally, and in cases of spontaneous abortion, the lack of neuropathological abnormalities may not exclude the fact that pregnancy loss could be the outcome of an underlying condition and that could also bias microglial findings linked to death. To date, most neuropathological studies have focused on predominantly qualitative methods to assess microglial populations in human embryonic/fetal tissues and with immunological (CD45, MHCII, CD68, IBA-1) or histochemical markers (RCA-1) that do not offer the possibility to distinguish between microglial cells and macrophages. This is likely to be a challenge for the field as development is highly dynamic, and we do not have specific markers to distinguish between cell lineages. New methods are needed to help identify a possible developmental microglial signature in the like of RNA-sequencing and single cell sorting methods from embryonic/fetal human tissue. To the author’s knowledge, only one study has emerged on this topic (102). At present, these tools are being used to identify enriched genes in human adult microglia against perivascular macrophages, for example, and to distinguish human microglial cells from rodent microglia (9, 10). Quantitative methods to assess microglial numbers and morphology in sections are needed to calculate the degree of proliferation, apoptosis, and to establish the microglial/macrophage marker developmental profile. These are much more likely to support our understanding of the regional heterogeneity of microglial cells during development and the dynamics of this population. Furthermore, the relevant mathematical models applied to these data would allow for covariate correction such as the post-mortem interval, brain weight, gestational age, gender, and anatomical location in order to reveal main effects on microglial numbers as well interactions between these independent variables. Significant advances in the field would be made with tightly controlled tissue samples from elective terminations and well-powered sample sizes in addition to emphasis on the previously mentioned points.

Finally, microglia feature as part of the pathophysiological signature of various neurodevelopmental disorders such as schizophrenia (5), intellectual disability (103), autism (104), and Rett’s syndrome (105). Therefore, understanding their precise dynamics and how they contribute to altering brain networks is likely to help us design therapeutic strategies to enhance their activity, limit the damage that could be caused by their activation, or use them as diagnostic biomarkers in disease.

## Conclusion

In the past decade, the field of study of microglia has rapidly expanded, and although rodent studies provide the opportunity to investigate how these cells populate the brain, develop, and proliferate, studies on the developing human brain are needed, particularly as the human and rodent adult brains have different microglial signatures and regional distributions. It remains unclear how these differences contribute to function and further studies correlating anatomy with function are needed.

## Author Contributions

DAM and DG-N conceived the structure and content of the manuscript. DAM wrote the manuscript and prepared the figures. DAM and DG-N drafted the manuscript.

## Conflict of Interest Statement

The authors declare that the research was conducted in the absence of any commercial or financial relationships that could be construed as a potential conflict of interest.
